# Gastroenterological Cancer and Immunotherapy

**DOI:** 10.1155/2018/4697670

**Published:** 2018-07-05

**Authors:** Xiaoping Wang, Yixin Eric Yang, Lintao Jia, Qi Chen

**Affiliations:** ^1^Department of Pathology, Shaanxi University of Chinese Medicine, Xianyang, Shaanxi, China; ^2^School of Natural Sciences, College of Natural, Applied and Health Sciences, Kean University, Union, NJ, USA; ^3^Department of Molecular Biology, Fourth Military Medical University, Xi'an, Shaanxi, China; ^4^Department of Pharmacology, Toxicology and Therapeutics, University of Kansas Medical Center, KS, USA

Gastroenterological cancers are the most common malignant tumors with the highest mortality in the world [1]. With the recent improvement in immunology, novel potential therapeutic molecules and immune cells for gastroenterological cancers have been recognized [2, 3]. However, there remains a lot to be learned about the function of immune molecules or cells for cancers. In the last few decades, immunotherapy has become a well-established strategy and found several applications in clinics or under clinical trials [4, 5]. Now, numerous forms of immunotherapeutic approaches are being explored for gastroenterological cancers. It is beneficial to exert the effects of the potential therapeutic molecules or immunological molecules or cells for gastroenterological cancers and try to avoid their side effects in the body.

Recently, new insights into the mechanisms involved in the immunotherapy for gastroenterological cancers have been explored. Novel biological effects of known therapeutic immunological molecules or cells against gastroenterological cancers have attracted much attention [6–10].

Immune checkpoints are involved in regulation of antigen recognition of T-cell receptor by costimulatory or inhibitory signaling transduction in the immune system. Immune checkpoint blockade therapy achieves great success in treating many types of cancers [6–8]. It targets T-cell regulatory pathways to enhance anticancer immune response. In recent years, cytotoxic T lymphocyte protein-4 (CTLA-4) and programmed cell death protein-1 (PD-1) have shown promise as novel therapeutic targets in some cancers [7–10]. Cytotoxic T lymphocyte protein-4 (CTLA-4) and programmed cell death protein-1 (PD-1) are immune checkpoints that inhibit the T-cell response, which provide the escape mechanism of the tumor cells to T-cell antitumor activity [8, 9]. The B7-H1, also known as PD-L1, in positive tumors interacts with its receptor PD-1, which leads to the inhibition of T-cells migration, proliferation, resulting in an antiapoptotic signal, preventing overactivation of the immune system, and escaping from destruction [8–10]. CTLA-4 is an immune checkpoint receptor expressed on regulatory T (Treg) cells and recently activated conventional T-cells [11, 12]. It is a negative regulator of T-cell activation. The anti-CTLA-4 antibody can blockade CTLA-4 interaction with B7 and prevents the inhibitory signal [12]. Targeting CTLA-4 with a human anti-CTLA-4 antibody has demonstrated therapeutically success in the treatment of melanoma [12, 13]. Then blockade of CTLA-4 may be a promising new approach to cancer therapy and constitutes a novel approach to induce host responses against tumors. It could downregulate the immune system and produce durable anticancer responses.

The better understanding of T-cell biology and genetic engineering allows us to modify T-cells by associating a synthetic molecule and infusing them into tumor tissue to enhance the immune response against malignant lesion [14]. Genetically engineered T-cells can specifically target cancer cells to eradicate tumor burden through a T-cell receptor or chimeric antigen receptors (CARs). CARs, also known as chimeric immunoreceptors, are engineered recombinant receptors with an intracellular signaling domain consisting of T-cell receptor-CD3-*ξ* domain and an extracellular single-chain variable antibody fragment [15]. CARs can directly bind to tumor-associated antigens, carbohydrates, or glycolipids. The antigens overexpressed on solid tumor cells but with limited or no expression on normal cells can be promising targets for CAR T-cell therapy.

Ongoing and future research will probably provide more efficient immunological molecules or cells for preventing and treating gastroenterological cancers.

This special issue encompasses cutting-edge research and review articles focusing on the role of the potential therapeutic and immunological molecules or cells in gastroenterological cancers. It includes 4 novel research articles and 3 reviews describing the advance of immunotherapy for gastric, colonic, and pancreatic cancers, summarized as follows.

***(1) Immunotherapy for Gastric Cancer***

In the review article titled “Immunotherapy in Advanced Gastric Cancer: An Overview of the Emerging Strategies”, H. Magalhães et al. summarized that the recent molecular characterization in gastric cancer will help us to better select patients who might benefit from immune checkpoint inhibitors and other agents.

There are encouraging results with agents that target programmed death 1 (PD-1) and its ligands in gastric cancer; however, more trials are needed to identify predictive and prognostic biomarkers to select patients most appropriate for this treatment. In this review, the authors explore the current evidence supporting the use of immunotherapy in advanced GC.

In the research article titled “Establishment of a Model of Microencapsulated SGC7901 Human Gastric Carcinoma Cells Cocultured with Tumor-Associated Macrophages”, the authors established a model of microencapsulated SGC7901 human gastric cancer cells and evaluated the effects of coculturing spheres with tumor-associated macrophages (TAMs). SGC7901 cells were encapsulated in alginate-poly lysine-sodium alginate (APA) microcapsules using an electrostatic droplet generator. MTT assays showed that the numbers of microencapsulated cells were highest after culturing for 14 days. Metabolic curves showed consumption of glucose and production of lactic acid by day 20. Immunocytochemistry confirmed that Proliferating Cell Nuclear Antigen (PCNA) and Vascular Endothelial Growth Factor (VEGF) were expressed in microencapsulated SGC7901 cells on days 7 and 14. The expression of PCNA was observed outside of spheroids; however, VEGF was found in the entire spheroids. PCNA and VEGF were increased after being cocultured with TAMs. Matrix metalloproteinase-2 (MMP-2) and matrix metalloproteinase-9 (MMP-9) expressions were detected in the supernatant of microencapsulated cells cocultured with TAMs but not in microencapsulated cells. The study confirms that coculturing of the microencapsulated GC cells with TAMs can promote PCNA, VEGF, MMP-2, and MMP-9 expressions of the GC cells.

***(2) Anesthesia on Immune Function in Colorectal Cancer Patients***

According to the review article titled “The Effect of Anesthesia on the Immune System in Colorectal Cancer Patients”, colorectal cancer (CRC) is the key leading cause of high morbidity and mortality worldwide. Surgery excision is the most effective treatment for CRC. However, stress caused by surgery response can destroy the body's immunity and increase the likelihood of cancer dissemination and metastasis. Anesthesia is an effective way to control the stress response, and recent basic and clinical research has shown that anesthesia and related drugs can directly or indirectly affect the immune system of colorectal cancer patients during the perioperative period. Thus, these drugs may affect the prognosis of CRC surgery patients.

This review is intended to summarize currently available data regarding the effects of anesthetics and related drugs on perioperative immune function and postoperative recurrence and metastasis in CRC patients. Determining the most suitable anesthesia for patients with CRC is of the utmost importance.

The editors expect this special issue to be of interest to the readers of recent advances in immunotherapy for gastroenterological cancers and anticipate that it will help researchers in making further progress in the understanding and development of immunotherapy for gastroenterological cancers.

***(3) Immunotherapy and Diagnostic Approaches for Pancreatic Cancer***

According to the review article titled “Combination Immunotherapy Approaches for Pancreatic Cancer Treatment”, immunotherapies have been evaluated in clinical trials and received great success in many types of cancers in last decades. However, they have very limited success in treating pancreatic cancer. As pancreatic cancer poorly responds to many single immunotherapeutic agents, combination immunotherapy was introduced to improve efficacy. The combination therapies hold great promise for enhancing immune responses to achieve better therapeutic effects. This review summarizes the existing and potential combination immunotherapies for the treatment of pancreatic cancer.

The research article titled “A Comparison of Endoscopic Ultrasound-Guided Fine-Needle Aspiration and Fine-Needle Biopsy in the Diagnosis of Solid Pancreatic Lesions” indicated that endoscopic ultrasound (EUS) guided fine needle aspiration (FNA) is the method of choice for sampling pancreatic lesions. This study compares the diagnostic accuracy and safety of fine needle biopsy (FNB) using a novel core needle to FNA in solid pancreatic lesions. A retrospective review of patients in whom EUS FNA or FNB was performed for solid pancreatic lesions was conducted. Diagnostic performance was calculated based upon a dual classification system. The results indicated that FNA and FNB have comparable sensitivity and diagnostic accuracy. FNB required fewer passes.

***(4) Immune Balance and Ulcerative Colitis***

The research article titled “EGCG Maintains Th1/Th2 Balance and Mitigates Ulcerative Colitis Induced by Dextran Sulfate Sodium through TLR4/MyD88/NF*κ*B Signaling Pathway in Rats” aimed to observe the protective effect of epigallocatechin gallate (EGCG) on dextran sulfate sodium- (DSS) induced ulcerative colitis in rats and to explore the roles of TLR4/MyD88/NF*κ*B signaling pathway in the protective effect of EGCG. Rat models of ulcerative colitis were established by giving DSS. EGCG was given to assess disease activity index. The results showed that EGCG improved the intestinal mucosal injury in rats with ulcerative colitis, inhibited production of inflammatory factors, maintained the balance of Th1/Th2, and reduced the expression of TLR4, MyD88, and NF*κ*B. After TLR4 antagonism, the protective effect of EGCG on intestinal mucosal injury was weakened in rats with ulcerative colitis, and the expressions of various inflammatory factors were upregulated. Therefore, EGCG can inhibit the intestinal inflammatory response by reducing the severity of ulcerative colitis and maintaining the Th1/Th2 balance through the TLR4/MyD88/NF*κ*B signaling pathway, which provides theoretical basis for development of target therapy for ulcerative colitis.

The research article titled “Decreased Breg/Th17 Ratio Improved the Prognosis of Patients with Ulcerative Colitis” by X. Bing et al. aimed to investigate the effects of regulatory B (Breg) cells and T helper 17 (Th17) cells on pathogenesis of ulcerative colitis, explore the clinical significance of Breg/Th17 ratio on the prognosis of ulcerative colitis, and provide the theoretical basis for the targeted therapy, diagnosis, and prognosis of the disease. Peripheral blood and colonic mucosa were collected from patients with ulcerative colitis. The colonic mucosa of ulcerative colitis patients presented massive inflammatory cell infiltration and hemorrhagic necrosis. The number of Breg cells and Th 17 cells, the gene expressions of IL-10 and ROR*γ*T, and serum levels of IL-10 and IL-17 all increased in peripheral blood. Compared with nonremission group, the remission group showed that the percentage of Breg cells reduced, the percentage of Th 17 cells increased, and thus the B10/Th17 ratio was significantly decreased in peripheral blood. In addition, serum IL-10 levels diminished, IL-17 levels increased, and thus IL-10/IL-17 ratio was remarkably reduced in remission group. B10/Th17 ratio and IL-10/IL-17 ratio were positively correlated with the severity of disease. Therefore, Breg and Th17 cells participate in the occurrence and development of ulcerative colitis. B10/Th17 ratio and IL-10/IL-17 ratio can be used as prognostic markers for ulcerative colitis. This provides a theoretical basis for design of targeted treatment and prognosis assessment of the disease.

*Xiaoping Wang* *Yixin Eric Yang* *Lintao Jia* *Qi Chen*
